# Triceps and Subscapular Skinfold Thickness Percentiles and Cut-Offs for Overweight and Obesity in a Population-Based Sample of Schoolchildren and Adolescents in Bogota, Colombia

**DOI:** 10.3390/nu8100595

**Published:** 2016-09-24

**Authors:** Robinson Ramírez-Vélez, Mario Ferney López-Cifuentes, Jorge Enrique Correa-Bautista, Katherine González-Ruíz, Emilio González-Jiménez, Diana Paola Córdoba-Rodríguez, Andrés Vivas, Hector Reynaldo Triana-Reina, Jacqueline Schmidt-RioValle

**Affiliations:** 1 Centro de Estudios para la Medición de la Actividad Física «CEMA», Escuela de Medicina y Ciencias de la Salud, Universidad del Rosario, Bogota DC 111221, Colombia; marlop26@gmail.com (M.F.L.-C.); jorge.correa@urosario.edu.co (J.E.C.-B.); 2 Grupo de Ejercicio Físico y Deportes, Vicerrectoria de Investigaciones, Universidad Manuela Beltrán, Bogota DC 110231, Colombia; katherine.gonzalez@docentes.umb.edu.co (K.G.-R.); jose.vivas@docentes.umb.edu.co (A.V.); 3 Departamento de Enfermería, Facultad de Ciencias de la Salud Avda, De la Ilustración, s/n, (18016), Universidad de Granada, Granada 18071, Spain; emigoji@ugr.es (E.G.-J.); jschmidt@ugr.es (J.S.-R.); 4 Grupo CTS-436, Adscrito al Centro de Investigación Mente, Cerebro y Comportamiento (CIMCYC), Universidad de Granada, Granada 18071, Spain; 5 Grupo de Alimentos, Nutrición y Salud, Facultad de Ciencias, Pontifica Universidad Javeriana, Bogota DC 110231, Colombia; d.cordoba@javeriana.edu.co; 6 Grupo GICAEDS, Facultad de Cultura Física, Deporte y Recreación, Universidad Santo Tomás, Bogota DC 110311, Colombia; hectortriana@usantotomas.edu.co

**Keywords:** skinfold thickness, percentile curves, children and adolescents, obesity

## Abstract

The assessment of skinfold thickness is an objective measure of adiposity. The aims of this study were to establish Colombian smoothed centile charts and LMS L (Box–Cox transformation), M (median), and S (coefficient of variation) tables for triceps, subscapular, and triceps + subscapular skinfolds; appropriate cut-offs were selected using receiver operating characteristic (ROC) analysis based on a population-based sample of children and adolescents in Bogotá, Colombia. A cross-sectional study was conducted in 9618 children and adolescents (55.7% girls; age range of 9–17.9 years). Triceps and subscapular skinfold measurements were obtained using standardized methods. We calculated the triceps + subscapular skinfold (T + SS) sum. Smoothed percentile curves for triceps and subscapular skinfold thickness were derived using the LMS method. ROC curve analyses were used to evaluate the optimal cut-off point of skinfold thickness for overweight and obesity, based on the International Obesity Task Force definitions. Subscapular and triceps skinfolds and T + SS were significantly higher in girls than in boys (*p* < 0.001). The ROC analysis showed that subscapular and triceps skinfolds and T + SS have a high discriminatory power in the identification of overweight and obesity in the sample population in this study. Our results provide sex- and age-specific normative reference standards for skinfold thickness values from a population from Bogotá, Colombia.

## 1. Introduction

The prevalence of overweight and obesity has become a public health problem worldwide [1]. International organizations [2,3] and previous epidemiological cross-sectional studies [4,5] have suggested that individuals with a large accumulation of excess body fat are at greater risk of the development of adverse health consequences, including hypertension, cardiovascular disease, metabolic disorders, osteoarthritis, gallbladder stone disease and asthma, as well as multiple malignancies [2,3,4,5,6,7]. To estimate the magnitude of this problem, direct indicators were used to assess various anthropometric indicators, such as body mass index (BMI) [6], waist circumference (WC) [7], and double thicknesses of skin and subcutaneous fat, measured as skinfold thickness [8]. Skinfold thickness measurements are widely used to assess body fat because the measurements are non-invasive, simple, and less expensive than laboratory-based techniques [9]; however, standardization and experience are required to achieve precise measurement [9,10].

There are a number of cross-sectional studies showing that high subcutaneous fat in youth is independently associated with higher cardio-metabolic risk [11,12,13]. In addition, longitudinal studies have shown that a healthy body composition in childhood and adolescence is associated with a healthier cardio-metabolic profile later on in life [14,15]. These findings have been replicated in clinical adult populations with diabetes mellitus, hypertension, metabolic syndrome, and several types of cancer [16,17]. The two most frequently taken skinfold measurements are from triceps and subscapular sites [9,18]. Expert panels from the USA suggest measurement of these two skinfolds as a part of in-depth medical assessments for adolescents with increased BMI [19]. However, Kromeyer-Hauschild et al. [18] found that the additional information provided by skinfolds varies substantially according to BMI levels.

Sex- and age-specific normative values for two skinfolds in youth have been published [18,20,21,22]. However, the majority of the published skinfold thickness reference values are for schoolchildren from high-income countries in North America [20] and Europe [18,21,22]. There is a scarcity of reference values for children, using harmonized measures of body composition, in Latin America [23,24] and other low-middle income countries (LMICs) undergoing rapid epidemiologic and nutrition transitions [25], making it impossible to evaluate secular trends within these regions and identify high risk groups for which risk reduction interventions should be prioritized. In particular, no population-based studies have been conducted to assess skinfold thickness for youths living in Bogotá, Colombia.

The aims of this study were to establish Colombian smoothed centile charts and LMS (L (Box–Cox transformation), M (median), and S (coefficient of variation)) tables for triceps, subscapular, and triceps + subscapular skinfolds, to develop appropriate cut-offs using receiver operating characteristic analysis, based on a population-based sample of 9- to 17-year-old children in Bogotá, Colombia, and to compare them with international studies.

## 2. Materials and Methods 

### 2.1. Study Population 

In Colombia, measures of weight and physical activity have been added to youth health monitoring systems by the government [26] and research institutions. Recently (2015), physical fitness assessment was added to the FUPRECOL study (in Spanish, Asociación de la Fuerza Prensil con Manifestaciones de Riesgo cardiovascular Tempranas en Niños y Adolescentes Colombianos). The FUPRECOL study seeks to establish the general prevalence of cardiovascular risk factors (anthropometric, metabolic, and genetic markers) in the study population (children and adolescents aged 9 to 17.9 years living in Bogotá, Colombia) [27,28] and to examine the relationships between physical fitness levels, body composition, and cardio-metabolic risk factors.

The FUPRECOL study assessments were conducted during the 2014–2015 school year. A total of 10,000 students were considered for physical fitness evaluation from all regions and 20 municipalities (‘localidades’) in the capital district of Bogotá. The sample consisted of children and adolescents (boys *n* = 4500 and girls *n* = 5500). We removed cases due to missing (*n* = 102, 1.2%) or erroneous data entry (*n* = 86, 0.8%) and those which had neither age nor a valid date of birth recorded (*n* = 194, 1.9%), limiting the analytical sample to 9618 students (boys *n* = 4253, 44.2% and girls *n* = 5365), aged 9–17.9 years. All children and adolescents were of low-middle socioeconomic status determined by the System of Identifying Potential Beneficiaries of Social Programs (SISBEN for its Spanish initials, 1–6 on a scale defined by the Colombian authorities). It takes into account sociodemographic characteristics (family composition, employment status, family income, and educational level), living conditions (construction type and materials), and access to public utilities (sewer, electricity, potable water, and garbage collection). Households with SISBEN levels 1–3 are the most vulnerable and targeted in social programs. SISBEN levels 4–6 is considered the least vulnerable sector of society. Participants were enrolled in public elementary and high schools (grades 5 through 11) in the capital district of Bogotá, Cundinamarca Department in the Andean region. This region is located at approximately 4°35′56″ N 74°04′51″ W, and is at an elevation of approximately 2625 metres (min: 2500; max: 3250) above sea level. Bogotá is considered an urban area, with approximately 7,862,277 inhabitants [29]. A convenience sample of volunteers was included and grouped by sex and age in one-year increments (a total of nine groups). Power calculations were based on the mean of overweight and obesity from the first 200–400 participants in the ongoing data collection (range 26–32 kg/m^2^), with a group standard deviation (SD) of approximately 5.2 kg/m^2^. The significance level was set to 0.05, and the required power was set to at least 0.80. The sample size was estimated to be approximately 250 to 500 participants per group. Individuals with systemic infections, endocrine disorders, cardiovascular disease, syndromic obesity, psychiatric disorders, pregnancy, asthma, use of any prescribed drugs, or any active use of illegal or illicit drugs, or was unable to participate in this study due to a physical impairment, were excluded from this investigation.

### 2.2. Data Collection

Anthropometric variables were measured by six evaluators certified by the International Society for the Advancement of Kinanthropometry (ISAK) in accordance with the ISAK guidelines [30]. Variables were collected at the same time in the morning, between 7:00 and 10:00 a.m., following an overnight fast. The body weights of the subjects were measured when the subjects were in their underwear and were not wearing shoes, using electronic scales (Tanita^®^ BC544, Tokyo, Japan) with a low technical error of measurement (TEM = 0.510%). Height was measured using a mechanical stadiometer platform (Seca^®^ 213, Hamburg, Germany; TEM = 0.019%). BMI was calculated as the body weight in kilograms divided by the square of height in metres. BMI was classified as underweight, normal weight, overweight, or obese using the International Obesity Task Force (IOTF) criteria [3]. WC was measured at the midpoint between the last rib and the iliac crest using a tape measure (Ohaus^®^ 8004-MA, Parsippany, NJ, USA; TEM = 0.086%). Triceps and subscapular skinfold thickness was measured by highly trained and standardized technicians, following recommended protocols [20]. Skinfold thickness was measured from the left side of the body to the nearest 0.1 mm, using a Holtain skinfold caliper (Holtain Ltd., Crymych, UK), at the following sites: (1) triceps, halfway between the acromion process and the olecranon process; and (2) subscapular, approximately 20 mm below the tip of the scapula, at an angle of 45° to the lateral side of the body. TEM was 3.248% for the triceps skinfold and 3.839% for the subscapular skinfold. The protocol of the reliability study proposed was taken in a subsample (*n* = 229, median age = 12.8 ± 2.4 years, 46.2 ± 12.4 kg, 1.50 ± 0.1 m, 19.9 ± 3.1 kg/m^2^). Every observer measured each child three consecutive times within 1 h for intra-observer assessment, while an inter-observer reliability investigation was performed on a separate day. The guidelines for the conduct of each measurement were deposed in standard operation procedures, which were compiled in a fieldwork manual and provided to all evaluators. The corresponding intra-observer technical error (reliability) of the measurements was 0.976% for the triceps skinfold and 0.979% for the subscapular skinfold [20]. Mean values were obtained from the three measurements, and the triceps + subscapular skinfold (T + SS) index was calculated [31]. The data were recorded on paper by FUPRECOL evaluators.

Finally, international studies were included for comparison if they explicitly reported descriptive triceps and subscapular skinfold thickness reference data at the sex, age, or country level. Study participants must have been apparently healthy (free from known disease or injury) and 9–17 years old.

### 2.3. Ethics Statement

The Review Committee for Research on Human Subjects at the University of Rosario (code no. CEI-ABN026-000262) approved all study procedures. A comprehensive verbal description of the nature and purpose of the study, and any experimental risks, was given to participants and their parents or guardians; this information was also sent to parents or guardians by mail. Written informed consent was obtained from parents and subjects before participation in the study. The protocol was in accordance with the latest revision of the Declaration of Helsinki and current Colombian laws governing clinical research on human subjects (Resolution 008430/1993 Ministry of Health).

### 2.4. Statistical Analysis 

Anthropometric characteristics of the study sample are presented as means and SD. Normality of selected variables was verified using histograms and Q-Q plots. Differences were analyzed using two-way analysis of variance (ANOVA) or chi-square test (χ^2^) in order to explore sex and age differences. Smoothed and specific curves for each age were obtained via a penalized maximum likelihood with the following abbreviations: (1) L (Box–Cox transformation); (2) M (median); and (3) S (coefficient of variation) [32]. The LMS method assumes that the outcome variable has a normal distribution after a Box–Cox power transformation is applied using the LMS method implemented in LMSChartMaker Pro Version 2.54 (Medical Research Council, London, UK. The appropriate number of degrees of freedom was selected on the basis of deviance, Q-tests, and worm plots, following the suggestions of Royston and Wright [33]. The 3rd, 10th, 25th, 50th, 75th, 90th, and 97th smoothing percentiles were chosen as age- and gender-specific reference values. The relation between skinfold thickness (triceps, subscapular, and T + SS) and overweight/obesity as defined by IOTF [34] was investigated with receiver operating characteristic (ROC) curves. Cut-off values were derived mathematically from the ROC curves using the point on the ROC curve with the lowest value for the formula (1 − sensitivity)^2^ + (1 − specificity)^2^. The positive and negative likelihood ratios, (LR) (+) and (LR) (−), were also determined. We used SPSS V. 21.0 software for Windows (SPSS, Chicago, IL, USA) for all but the LMS method calculations. Statistical significance was set at *p* < 0.05.

## 3. Results

### 3.1. Descriptive Characteristics

Descriptive statistics for each sex are shown in Table 1. All of the anthropometric variables, except weight and height, were higher in girls than in boys (*p* < 0.01). The chi-square tests showed that normal weight was higher in boys than in girls (*p* < 0.01).

### 3.2. Normative Skinfold Thickness Values 

**Figure 1:**
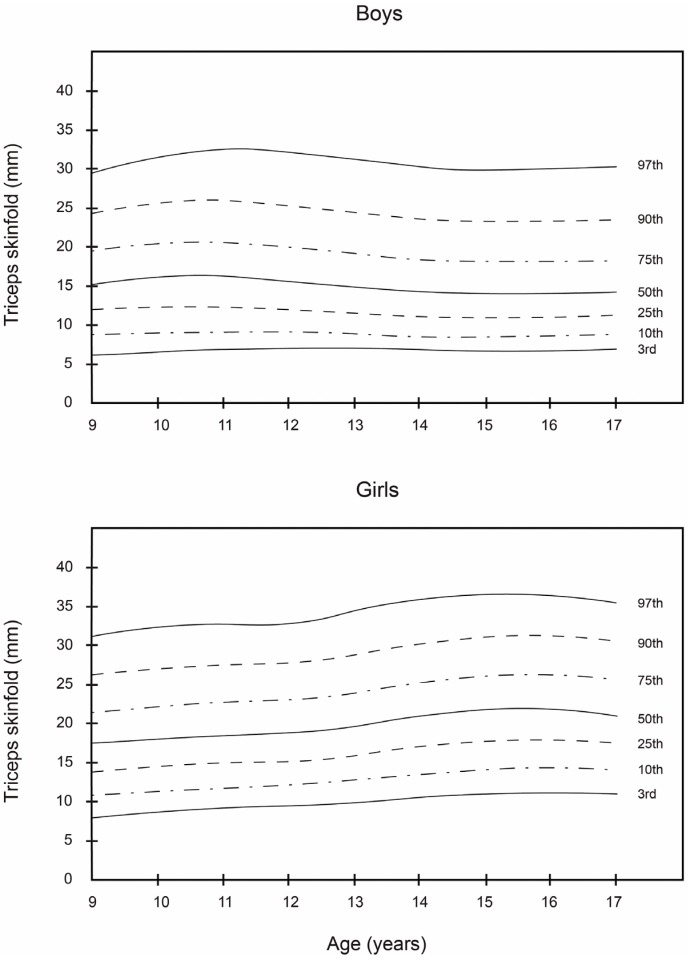
Smoothed percentile curves for skinfolds (in mm) for boys and girls across ages, from Bogota, Colombia: Triceps.

**Figure 2:**
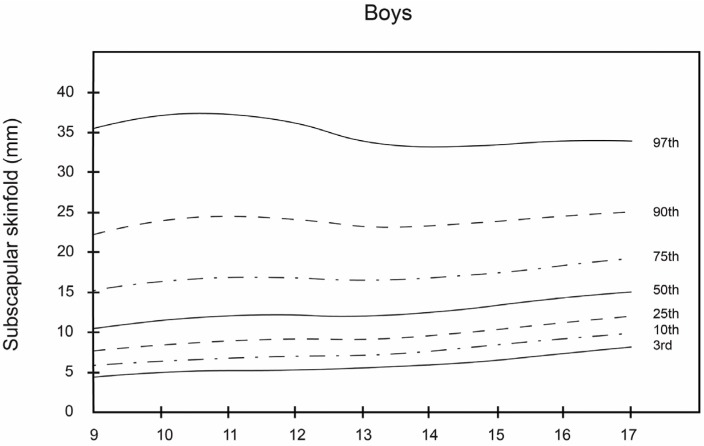

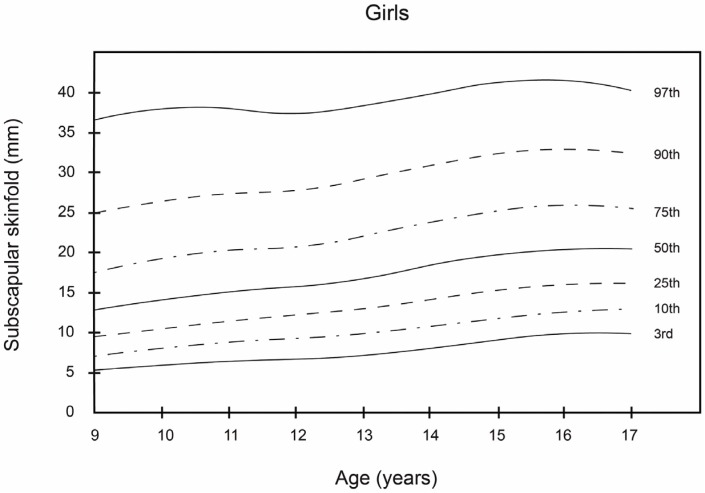
Smoothed percentile curves for skinfolds (in mm) for boys and girls across ages, from Bogota, Colombia: Subscapular.

**Figure 3:**
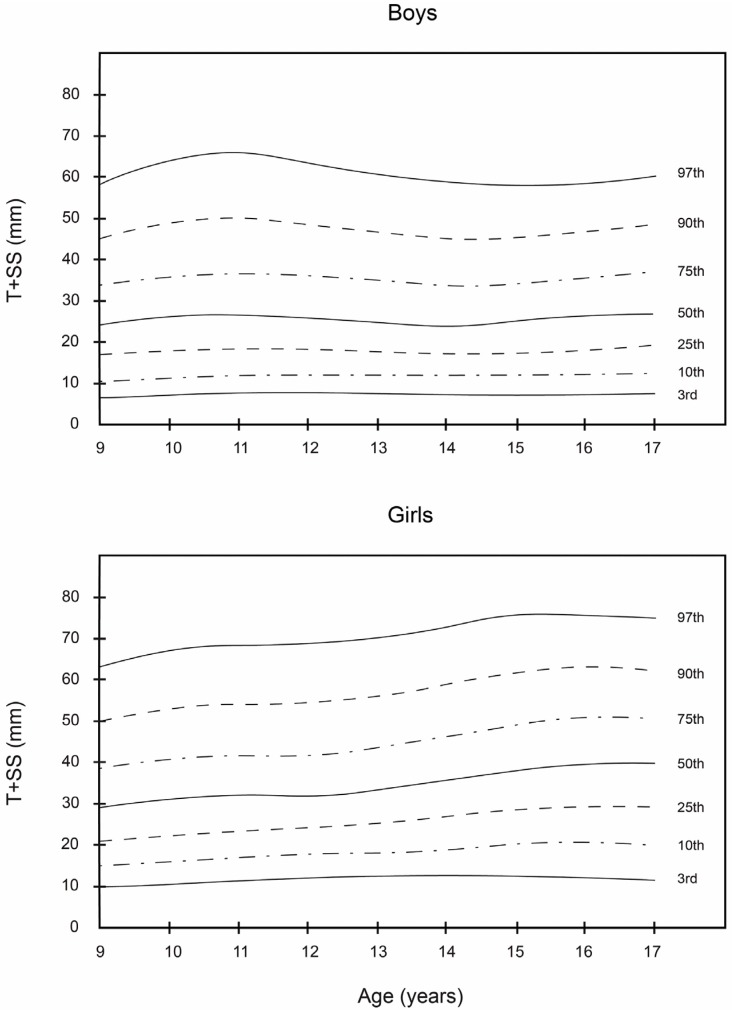
Smoothed percentile curves for skinfolds (in mm) for boys and girls across ages, from Bogota, Colombia. Triceps + Subscapular (T + SS).

Smoothed LMS curves (3rd, 10th, 25th, 50th, 75th, 90th, and 97th percentile) for boys’ and girls’ skinfolds (subscapular, triceps and T + SS) are shown in Table 2, Table 3 and Table 4. The equivalent numerical values are available in Figure 1, Figure 2 and Figure 3.

Together, these data show that girls had higher subscapular, triceps, and T + SS values at all ages compared with boys. In boys, the 50th percentile of triceps, subscapular, and T + SS ranged from 10.0 to 15.0 mm, 10.0 to 15.0 mm, and 23.0 to 28.0 mm, respectively. In girls, the 50th percentile ranged from 14.0 to 22.0 mm, 12.0 to 21.0 mm, and 28.0 to 42.0 mm, respectively. In all age classes, girls had a higher 50th percentile values than boys. In boys, triceps skinfold peaked at 11 to 13.9 years and subsequently decreased, whereas, in girls, the triceps skinfold steadily increased. The subscapular skinfold rose steadily in both sexes between 9 and 14 years of age. The percentile distribution was more dispersed towards higher values mainly for T + SS.

ROC analysis showed that triceps, subscapular, and T + SS could detect IOTF overweight and obesity (Table 5). For example, in the overweight category in boys aged 9–11.9, the cut-off value of 18.1 mm for triceps skinfolds provided a sensitivity of 82.3%, an LR (+) value of 4.06, specificity of 71.9%, and an LR (−) value of 0.34. In girls aged 9–11.9, the cut-off value of 20.1 mm for triceps skinfolds provided a sensitivity of 67.0%, an LR (+) value of 3.72, specificity of 82.0%, and an LR (−) value of 0.40. For the obesity category in boys aged 15–17.9, the cut-off value of 21.8 mm for subscapular skinfolds provided a sensitivity of 70.6%, an LR (+) value of 4.94, specificity of 85.7%, and an LR (−) value of 0.34. In girls aged 15–17.9, the cut-off value of 28.7 mm for subscapular skinfolds provided a sensitivity of 63.2%, an LR (+) value of 5.06, specificity of 87.5%, and an LR (−) value of 0.42.

An ROC curve for T + SS was also obtained (Table 5). In the overweight category in boys aged 9–11.9, the cut-off value of 34.6 mm for T + SS provided sensitivity of 70.5%, an LR (+) value of 7.42, specificity of 90.5%, and an LR (−) value of 0.33. In girls aged 12–14.9, the cut-off value was 43.1 mm, sensitivity 61.7%, LR (+) value 3.98, specificity 84.5%, and LR (−) 0.45. With respect to obesity in boys aged 15–17.9, a cut-off value of 48.6 mm was used. The sensitivity was 58.0%, LR (+) value 3.43, specificity 83.1%, and LR (−) 0.51. In girls aged 15–17.9, the cut-off value was 39.9 mm with sensitivity 76.5%, LR (+) value 4.11, specificity 81.4%, and LR (−) 0.29.

### 3.3. International Comparison 

Finally, comparisons with other studies of the 50th percentile for triceps and subscapular skinfolds (mm) from this study are presented in Table 6. Based on the raw non-adjusted data, we found that Bogotá boys and girls had higher triceps and subscapular skinfold values than their counterparts from Spain, the UK, Germany, and the USA.

## 4. Discussion

The results obtained in this study presented for the first time smoothed reference values for the triceps and subscapular skinfold thicknesses of a large, population-based sample of schoolchildren from Bogotá, Colombia. Although the boys had higher weight and height values, the girls had a higher BMI. In addition, a greater prevalence of normal weights among boys was shown, followed by a higher rate of overweight and obesity in girls. These results coincide with those obtained by Freedman et al. [35], who performed a prospective study of 6866 boys and girls, 5–17.9 years of age, in Louisiana (USA). In this research, the BMI values of the girls were considerably higher than those of the boys.

In contrast, our results differed somewhat from Aristizabal et al. [36]. According to their study of 232 schoolchildren in Medellín (Colombia), there was a higher prevalence of normal weight and overweight among girls, whereas higher values of obesity were detected among boys (as both BMI and *z*-score BMI). A possible explanation for this partial divergence in results could be the age difference between our sample population and theirs. More specifically, the schoolchildren in Medellín were younger than the children in our sample.

In regard to subcutaneous fat distribution, striking differences were observed in triceps and subscapular skinfold thickness, as well as in the sum of both values (T + SS). In fact, these values were significantly higher for girls in the study than for boys. This coincides with the findings of Aristizabal et al. [36] in Medellín (Colombia). Our results also agreed with those reported by Nagy et al. [37] for a sample of 16,228 boys and girls from different European countries (Sweden, Germany, Hungary, Italy, Cyprus, Spain, Belgium, and Estonia), where girls also had higher scores for triceps and subscapular skinfold thickness, as well as the sum of both (T + SS).

In the case of WC, there were significant differences between both sexes, with higher values for boys. These results agree with those of Hirschler et al. [38] in a population of Argentinian children, in which the boys were found to have a larger WC than the girls [38]. Since our data are not longitudinal, we do not know whether WC increases progressively with age. Nevertheless, modification of subcutaneous fat distribution over time is widely documented in the literature. More specifically, with age, subcutaneous fat tends to move from the periphery to the trunk, which increases the risk of cardiovascular disorders at an earlier age [39,40].

As can be observed, the smoothed LMS curves show higher values for triceps and subscapular skinfold thickness and T + SS for girls, regardless of age, in comparison with boys. These results are similar to those reported by Kromeyer-Hauschild et al. [18] for a population of 2132 boys and girls in the city of Jena (Germany). In this study, the girls in all age groups had the highest mean values for these skinfolds. In regard to the boys, the 50th percentile of the triceps and subscapular skinfold thickness and T + SS varied from 10.0 to 14.0 mm, from 10.0 to 15.0 mm, and from 23.0 to 28.0 mm, respectively. Among the girls, the 50th percentile for these same skinfold thicknesses ranged from 17.0 to 22.0 mm, from 12.0 to 21.0 mm, and from 28.0 to 42.0 mm, respectively.

As reported by Aristizabal et al. [36] and Addo and Himes [20], girls in this study had higher mean values for these skinfolds than boys in all age groups. Among boys, triceps skinfold reached a maximum value of 31 mm at the age of 13.9 years, after which it decreased. In contrast, for girls, the values of these skinfolds increased progressively. As for subscapular skinfold thickness, it increased steadily in both sexes from 9 to 14 years of age. Similar findings were reported in previous studies of schoolchildren, though in other geographic areas, namely Warsaw [41], Krakow [42], and Turkey [43]. In the case of T + SS, the percentage distribution of the values was more dispersed, with higher values for the girls. These results differed somewhat from those of Moreno et al. [21], who obtained higher T + SS values for boys in their study of 2160 Spanish adolescents, 13–18 years of age.

ROC analysis showed that triceps, subscapular, and T + SS had a high discriminating power to detect overweight and obesity. This coincided with Cickek et al. [44] and their study of a population of Turkish children and adolescents. In the overweight category for boys, 9–11.9 years of age, the cut-off value for the triceps skinfold was 18.1 mm, whereas for girls in the same age group the cut-off value was 20.1 mm. These results agreed with those of Brannsether et al. [45] and their study of 4606 Norwegian children, in which higher cut-off values were reported for girls in all age groups. Similar results were obtained by Khadilkar et al. [46], who studied a population of 13,375 schoolchildren in India.

In the obesity category for boys aged 15–17.9 years, the cut-off value for subscapular skinfold was 21.8 mm. In contrast, for girls of the same age, the cut-off value was considerably higher (28.7 mm). These results were similar to those of Kromeyer-Hauschild et al. [18] for a sample population of 213 school children in Jena (Germany). In our study, the overweight and obesity cut-off values of the subscapular skinfold were much higher for girls, which reflected that girls had a higher level of subcutaneous adiposity than boys.

In addition, the sensitivity of subscapular skinfold to detect overweight in body fat composition with respect to BMI is 49.4% in girls and 74.6% in boys. However, the specificity is 84.1% in girls and 70.6% in boys. Therefore, using the subscapular skinfold there is a significant percentage of girls who, despite having normal levels of fat, would be categorized in the group of excessive fat. Taking into account this observation, the criteria for selecting a cut-off value (i.e., accepting or rejecting) should be considered when sensitivity is above 80%. In fact, ROC curves represent the rate of true positives versus the rate of false positives and are used to determine a more precise cut-off. Furthermore, the cut-off point for T + SS in both the overweight and obesity categories (especially in the 15–17.9 age group) was higher for girls. This could be explained by the marked sexual dimorphism in regard to the development and accumulation of subcutaneous fat, which increased as the subjects’ age increased [47,48,49,50]. In the obesity category for boys 15–17.9 years old, the cut-off point of 39.9 mm for T + SS provides a sensitivity of 76.5% and specificity of 81.4%. In girls, the cut-off point of 53.7 mm provides a sensitivity of 67.4% and a specificity of 83.5%. The area under the ROC curve is also considered in the analysis of ROC curves. This area under the ROC curve is a measure of how well a parameter can distinguish between two diagnostic groups.

Cross-cultural comparisons were conducted between the triceps and subscapular skinfold data from this study in Colombia with those from Spain [18], Germany [21], the UK [22], and the USA [20]. These countries were selected to represent European and American regions, respectively, for comparison with our data for the South American region. This comparative study based on non-fitted raw data showed that both boys and girls in Bogotá had higher values for tricipital and subscapular skinfolds in all of the age groups than children of similar ages in Spain, the UK, Germany, and the USA. This signifies that this sample population of children and adolescents in Bogotá generally had higher levels of adiposity than similar samples in other studies in other countries. Children and adolescents from different ethnic populations vary in their rate of proportional growth and body fat patterning. Our own results indicate differences between ethnic groups for both triceps and subscapular skinfolds. This ethnic difference in body fat distribution has also been observed in adolescents and pre-pubertal children mainly from the South America region, again highlighting the importance of ethnicity-specific studies.

Evidently, this is an important public health problem in Colombia. For this reason, it is necessary to carry out new studies that will help to identify and control the factors leading to this higher level of adiposity among schoolchildren in Bogotá. Moreover, these results should serve as a wake-up call for the Colombian government, which needs to implement policies that will encourage healthier lifestyles among young people, such as regular physical exercise and a balanced diet from early childhood.

This study had some limitations. First, it includes participants from only a single region in Colombia; therefore, inferences for all Colombian children and adolescents should be made cautiously. This study includes participants from public schools in one city and thus the data are not fully representative of the full population either of the city or of the country. However, Bogotá is the largest urban centre in Colombia, comprising about 15% of the country’s population. It includes a mix of locally born residents and people born elsewhere. The FUPRECOL study was deployed in collaboration with the Bogotá District Education Department, which only has jurisdiction among public schools. However, the public system constitutes the majority of school offering in the city, with 85% of school-age children enrolled in the city public school system. Second, we have not considered the potential impact of recognized determinants, such as socioeconomic, dietary, and physical activity patterns and ethnic factors that modulate growth and levels of adiposity. Third, this study includes use of skinfold thickness as a measure of adiposity to define pediatric obesity from BMI and has not used expensive tools that are sometimes difficult to transport to the field (e.g., dual-energy X-ray absorptiometry, air displacement plethysmography, and bioelectrical impedance). Nonetheless, BMI is widely recognized as an appropriate tool to screen obese children and adolescents and to define overweight status, as a state of excessive weight relative to height, regardless of body composition. This could suppose an important advantage for BMI over dual-energy X-ray absorptiometry, bioelectrical impedance, or both. Fourth, the sensitivity and specificity of the skinfolds were calculated using BMI as the gold standard, and BMI is not a measure of body fat. In addition, when a diagnosis tool has low sensitivity, it has an important number of false negatives (in this case, girls that should be considered obese but are incorrectly classified as not obese by skinfold measurement). Finally, these curves should only be used for Colombian children. However, such limitations do not compromise the results obtained when validating our results.

This study also has various strong points that should be highlighted. The results presented, for the first time, smoothed reference values for triceps and subscapular skinfold thickness among a large, population-based sample of schoolchildren from Bogotá, Colombia. Additionally worth mentioning is the use of the LMS method to smooth the percentile values. This allowed an accurate description of body composition and the pattern of fat distribution in the sample population, as well as its variation, depending on sex and age.

## 5. Conclusions 

In summary, ROC analysis showed that subscapular and triceps skinfolds and T + SS have adequate discriminatory power in the identification of overweight and obesity in the sample population in this study. These results provide sex- and age-specific normative reference standards for skinfold thickness values from a population from Bogotá, Colombia.

## Figures and Tables

**Table 1:** Characteristics of among a population-based sample of children and adolescents in Bogota, Colombia (mean (SD) or frequencies).

Characteristics	Total (*n* = 9618)	Boys (*n* = 4253)	Girls (*n* = 5365)	*p* Value
Age (years)	12.8 (2.3)	12.9 (2.3)	12.8 (2.4)	0.708
Weight (kg)	44.6 (12.3)	45.0 (13.0)	44.8 (11.4)	0.01
Height (m)	1.49 (0.12)	1.50 (0.13)	1.47 (0.10)	<0.001
BMI (kg/m^2^)	19.7 (3.4)	19.3 (3.3)	20.3 (3.5)	<0.001
BMI (*z*-score)	−0.03 (0.51)	−0.02 (0.91)	0.43 (0.78)	<0.001
BMI status (%) *				
Underweight	29%	29%	31%	<0.001
Normal weight	49%	53%	45%	<0.001
Overweight	15%	13%	16%	<0.001
Obese	7%	6%	8%	<0.001
Waist circumference (cm)	65.6 (8.1)	65.0 (8.1)	66.3 (8.2)	<0.001
Triceps skinfold thickness (mm)	18.2 (6.6)	15.7 (6.2)	20.3 (6.2)	<0.001
Subscapular skinfold thickness (mm)	16.4 (8.0)	14.2 (7.5)	18.3 (8.0)	<0.001
Triceps + subscapular skinfolds (mm)	31.8 (15.1)	27.5 (13.8)	35.5 (15.0)	<0.001

Data are shown as mean (SD) or frequencies. Significant between-sex differences (ANOVA one way test *or* chi-square; * *p* < 0.001).

**Table 2:** Smoothed age- and sex-specific percentile values of triceps skinfold thickness (mm) among a population-based sample of children and adolescents in Bogota, Colombia.

	L	S	SD	P_3_	P_10_	P_25_	P_50_ (M)	P_75_	P_90_	P_97_
Boys										
9 to 9.9	−0.30	0.37	6.8	5.0	6.0	7.0	10.0	15.0	21.0	32.0
10 to 10.9	−0.31	0.39	6.5	7.0	9.0	12.0	15.0	21.0	25.0	32.0
11 to 11.9	−0.32	0.42	6.5	8.0	10.0	12.0	16.0	22.0	26.0	31.0
12 to 12.9	−0.33	0.46	6.3	7.9	9.5	12.0	15.0	20.0	25.0	31.1
13 to 13.9	−0.34	0.47	6.2	7.0	9.0	11.0	15.0	19.0	24.0	31.0
14 to 14.9	−0.35	0.48	6.1	7.0	8.0	11.0	14.0	18.0	23.2	29.9
15 to 15.9	−0.36	0.48	6.0	7.0	8.0	11.0	14.0	18.0	23.0	30.0
16 to 16.9	−0.37	0.49	5.8	7.0	9.0	11.0	14.0	17.0	22.7	29.0
17 to 17.9	−0.38	0.49	5.9	7.0	8.0	11.0	14.0	18.2	24.0	29.1
*Total*	*−0.33*	*0.43*	*6.2*	*7.0*	*9.0*	*11.0*	*14.8*	*19.0*	*24.0*	*30.0*
Girls										
9 to 9.9	−0.97	0.34	5.7	7.0	11.0	14.0	17.0	21.0	25.0	29.0
10 to 10.9	−0.97	0.36	6.0	10.0	12.0	15.0	18.0	22.0	26.0	32.0
11 to 11.9	−0.97	0.40	5.8	10.0	12.0	15.0	18.0	23.0	26.0	31.0
12 to 12.9	−0.98	0.40	5.8	10.0	12.0	15.0	18.0	22.0	27.0	33.0
13 to 13.9	−0.95	0.40	6.1	10.0	12.0	15.0	20.0	23.5	28.0	33.0
14 to 14.9	−0.95	0.40	6.4	11.4	14.0	17.0	22.0	25.0	30.0	34.0
15 to 15.9	−0.94	0.40	6.4	10.0	14.0	17.0	22.0	26.1	30.2	34.0
16 to 16.9	−0.94	0.39	5.8	12.0	15.0	18.0	22.0	26.0	30.0	34.0
17 to 17.9	−0.93	0.39	6.3	12.0	14.0	17.0	21.4	25.0	29.0	34.0
*Total*	*−0.95*	*0.38*	*6.2*	*10.0*	*12.9*	*16.0*	*20.0*	*24.0*	*28.0*	*33.0*

M: mean; SD: standard deviation; P: percentile; L: Box–Cox transformation; S: coefficient of variation.

**Table 3:** Smoothed age- and sex-specific percentile values of subscapular skinfold thickness (mm) among a population-based sample of children and adolescents in Bogota, Colombia.

	L	S	SD	P_3_	P_10_	P_25_	P_50_ (M)	P_75_	P_90_	P_97_
Boys										
9 to 9.9	−0.06	0.35	6.8	5.0	6.0	7.0	10.0	15.0	21.0	32.0
10 to 10.9	−0.05	0.39	7.4	5.0	6.0	8.0	11.0	16.0	24.0	32.0
11 to 11.9	−0.03	0.40	8.3	5.0	7.0	9.0	12.0	18.0	27.0	35.0
12 to 12.9	−0.01	0.41	8.1	6.0	7.0	8.0	12.0	17.0	25.0	35.0
13 to 13.9	−0.01	0.41	7.4	5.0	7.0	9.0	12.0	16.0	22.0	33.0
14 to 14.9	−0.01	0.41	6.9	6.0	7.0	9.0	12.0	16.0	23.0	30.0
15 to 15.9	−0.03	0.41	6.6	7.0	8.0	10.0	13.0	17.0	24.0	31.1
16 to 16.9	−0.05	0.39	6.4	8.0	9.0	12.0	14.0	18.0	23.0	32.1
17 to 17.9	−0.06	0.39	8.8	8.0	9.0	12.0	15.0	20.0	24.1	31.3
*Total*	*−0.03*	*0.38*	*7.5*	*6.0*	*7.0*	*9.0*	*12.0*	*17.0*	*24.0*	*33.0*
Girls										
9 to 9.9	−0.74	0.41	7.0	6.0	7.0	9.0	12.0	17.0	23.0	31.1
10 to 10.9	−0.71	0.40	7.9	7.0	8.0	10.0	14.0	20.0	26.0	35.0
11 to 11.9	−0.67	0.46	8.1	7.0	9.0	11.0	15.0	20.0	27.0	35.0
12 to 12.9	−0.58	0.47	7.8	7.0	9.0	12.0	15.0	21.0	27.5	36.0
13 to 13.9	−0.56	0.47	7.5	7.0	10.0	12.0	16.3	22.0	27.0	35.0
14 to 14.9	−0.45	0.47	7.7	9.0	11.0	14.0	18.0	24.0	30.0	36.0
15 to 15.9	−0.51	0.47	7.6	10.0	12.0	16.0	20.0	25.0	31.0	38.5
16 to 16.9	−0.47	0.46	7.7	10.0	12.0	16.0	21.0	26.0	32.0	38.0
17 to 17.9	−0.44	0.46	7.7	10.0	12.0	16.0	20.0	25.0	32.0	40.0
*Total*	*−0.57*	*0.44*	*8.0*	*7.0*	*10.0*	*12.0*	*17.0*	*23.0*	*29.0*	*36.0*

M: mean; SD: standard deviation; P: percentile; L: Box–Cox transformation; S: coefficient of variation.

**Table 4:** Smoothed age- and sex-specific percentile values of triceps + subscapular skinfolds (mm) among a population-based sample of children and adolescents in Bogota, Colombia.

	L	S	SD	P_3_	P_10_	P_25_	P_50_ (M)	P_75_	P_90_	P_97_
Boys										
9 to 9.9	−0.30	0.37	13.2	6.5	9.0	17.0	23.0	33.0	43.0	54.5
10 to 10.9	−0.31	0.39	14.0	7.0	13.9	19.0	26.0	35.0	49.2	60.0
11 to 11.9	−0.32	0.42	14.9	7.5	13.3	20.0	26.0	37.0	52.0	62.5
12 to 12.9	−0.33	0.46	14.9	7.0	10.5	18.0	26.0	35.0	49.0	64.2
13 to 13.9	−0.34	0.47	13.7	7.5	10.5	18.0	25.5	33.0	43.0	60.0
14 to 14.9	−0.35	0.48	13.2	7.5	10.5	18.0	25.0	31.0	44.1	56.0
15 to 15.9	−0.36	0.48	12.8	8.0	10.5	19.0	26.0	33.5	45.0	56.0
16 to 16.9	−0.37	0.49	13.1	8.0	10.2	21.0	27.0	34.0	45.0	55.1
17 to 17.9	−0.38	0.49	13.6	8.0	10.0	20.0	28.0	37.0	47.0	56.2
*Total*	*−0.33*	*0.43*	*13.8*	*7.5*	*10.5*	*18.0*	*26.0*	*34.0*	*46.0*	*59.0*
Girls										
9 to 9.9	−0.97	0.34	13.2	9.0	14.0	21.0	28.0	37.0	49.0	60.0
10 to 10.9	−0.97	0.36	14.3	12.0	16.9	23.4	30.0	41.0	51.2	66.0
11 to 11.9	−0.97	0.40	14.1	12.1	17.5	24.0	32.0	41.0	52.0	66.0
12 to 12.9	−0.98	0.40	14.2	11.5	15.7	24.0	32.0	41.0	52.0	68.0
13 to 13.9	−0.95	0.40	14.3	12.5	17.0	22.8	33.0	43.0	52.0	66.1
14 to 14.9	−0.95	0.40	14.8	15.0	20.0	27.0	36.0	46.0	57.0	71.6
15 to 15.9	−0.94	0.40	15.3	13.3	18.9	27.8	40.0	50.0	59.0	70.0
16 to 16.9	−0.94	0.39	15.2	12.4	18.5	32.0	42.0	50.0	61.0	70.0
17 to 17.9	−0.93	0.39	15.9	12.0	16.0	28.0	40.0	48.0	60.0	72.8
*Total*	*−0.95*	*0.38*	*15.0*	*12.0*	*17.0*	*24.0*	*34.0*	*45.0*	*55.0*	*68.0*

M: mean; SD: standard deviation; P: percentile; L: Box–Cox transformation; S: coefficient of variation.

**Table 5:** Receiver operating characteristic analysis to triceps, subscapular, and T + SS to detect IOTF overweight and obesity among Colombian children and adolescents aged 9–17.9 years.

	9–11.9 Years				12–14.9 Years				15–17.9 Years			
	Overweight		Obesity		Overweight		Obesity		Overweight		Obesity	
	Boys	Girls	Boys	Girls	Boys	Girls	Boys	Girls	Boys	Girls	Boys	Girls
Triceps skinfold												
AUC	0.87	0.83	0.88	0.87	0.83	0.78	0.82	0.82	0.82	0.74	0.79	0.80
Cut-off	18.1	20.1	22.1	21.1	18.3	21.2	21.6	24.6	16.2	25.5	21.5	27.5
J-Youden	0.54	0.49	0.61	0.53	0.46	0.40	0.48	0.49	0.45	0.34	0.41	0.39
Sensitivity (%)	71.9	67.0	71.7	77.4	65.9	66.9	63.7	67.5	74.6	49.4	55.9	53.7
Specificity (%)	82.3	82.0	88.9	75.9	80.1	72.9	84.3	81.5	70.6	84.1	84.8	84.9
LR (+)	4.06	3.72	6.46	3.21	3.31	2.47	4.06	3.65	2.54	3.11	3.68	3.56
LR (−)	0.34	0.40	0.32	0.30	0.43	0.45	0.43	0.40	0.36	0.60	0.52	0.55
Subscapular skinfold												
AUC	0.83	0.80	0.84	0.82	0.79	0.75	0.80	0.79	0.80	0.69	0.78	0.72
Cut-off	13.5	14.4	16.9	18.3	15.1	19.8	15.9	23.2	18.6	24.4	21.8	28.7
J-Youden	0.61	0.52	0.64	0.61	0.53	0.44	0.49	0.55	0.54	0.38	0.56	0.51
Sensitivity (%)	77.5	78.3	79.6	82.1	71.5	67.7	74.7	72.4	72.3	55.0	70.6	63.2
Specificity (%)	83.0	73.3	83.9	79.3	81.7	76.7	74.3	82.9	81.4	83.2	85.7	87.5
LR (+)	4.56	2.93	4.94	3.97	3.91	2.91	2.91	4.23	3.89	3.27	4.94	5.06
LR (−)	0.27	0.30	0.24	0.23	0.35	0.42	0.34	0.33	0.34	0.54	0.34	0.42
T + SS												
AUC	0.86	0.84	0.87	0.86	0.83	0.79	0.83	0.82	0.84	0.74	0.81	0.78
Cut-off	34.6	36.5	37.0	40.0	36.8	43.1	43.5	48.7	37.1	48.6	39.9	53.7
J-Youden	0.61	0.56	0.64	0.61	0.53	0.46	0.50	0.55	0.53	0.41	0.58	0.51
Sensitivity (%)	70.5	72.2	80.3	82.6	65.6	61.7	60.4	68.7	68.9	58.0	76.5	67.4
Specificity (%)	90.5	84.0	83.4	78.7	87.1	84.5	89.2	86.0	83.8	83.1	81.4	83.5
LR (+)	7.42	4.51	4.84	3.88	5.09	3.98	5.59	4.91	4.25	3.43	4.11	4.08
LR (−)	0.33	0.33	0.24	0.22	0.39	0.45	0.44	0.36	0.37	0.51	0.29	0.39

AUC: Area under curve; LR (+): Positive likelihood ratio; LR (−): Negative likelihood ratio; T + SS: Sum triceps + subscapular skinfold.

**Table 6:** Comparison of empirical triceps and subscapular skinfold thickness (mm) of the 50th percentile from international studies.

Study [Reference]	FUPRECOL Study	Spain [21]	Germain [18]	UK [22]	USA [20]	FUPRECOL Study	Spain [21]	Germain [18]	UK [22]	USA [20]
Year	2016	2007	2012	1974	2007	2016	2007	2012	1974	2007
*n*	9618	160	2132	19,700	32,783	9618	160	2132	19,700	32,783
Boys	Triceps skinfold (mm)					Subscapular skinfold (mm)				
9 to 9.9	10.0	-	11.0	8.1	9.1	10.0	-	6.0	5.2	5.2
10 to 10.9	15.0	-	11.4	8.5	9.5	11.0	-	6.5	5.6	5.5
11 to 11.9	16.0	-	14.0	8.9	9.7	12.0	-	7.3	6.0	5.8
12 to 12.9	15.0	-	14.0	9.0	9.5	12.0	-	8.0	6.4	6.1
13 to 13.9	15.0	11.4	13.0	8.8	9.1	12.0	8.9	8.0	6.6	6.4
14 to 14.9	14.0	11.0	13.3	8.2	8.6	12.0	9.5	10.0	6.8	6.8
15 to 15.9	14.0	10.3	-	7.8	8.3	13.0	9.4	-	7.4	7.3
16 to 16.9	14.0	9.9	-	8.0	8.1	14.0	9.6	-	8.0	7.9
17 to 17.9	14.0	10.9	-	9.0	8.1	15.0	10.6	-	9.0	8.6
Girls	Triceps skinfold (mm)					Subscapular skinfold (mm)				
9 to 9.9	17.0	-	12.5	10.8	11.6	12.0	-	7.0	6.6	6.5
10 to 10.9	18.0	-	12.0	11.1	12.2	14.0	-	7.0	7.0	7.1
11 to 11.9	18.0	-	15.0	11.5	12.8	15.0	-	9.0	7.8	7.8
12 to 12.9	18.0	-	15.5	11.8	13.4	15.0	-	11.0	8.8	8.5
13 to 13.9	20.0	15.9	15.1	12.0	14.1	16.3	12.3	10.0	9.4	9.2
14 to 14.9	22.0	15.3	16.0	13.0	14.9	18.0	11.5	11.0	10.2	10.0
15 to 15.9	22.0	15.6	-	14.2	15.7	20.0	11.7	-	11.4	10.7
16 to 16.9	22.0	16.6	-	15.5	16.5	21.0	12.2	-	12.0	11.4
17 to 17.9	21.4	16.8	-	16.0	17.2	20.0	12.2	-	12.4	12.0

## Abbreviations

The following abbreviations are used in this manuscript:

ANOVA	Analysis of variance
AUC	Area under curve
BMI	Body mass index
IOTF	The International Obesity Task Force
ISAK	International Society for the Advancement of Kinanthropometry
LMICs	Low-middle income countries
LMS	L (Box-Cox transformation), M (median), S (coefficient of variation)
LR (−)	Negative likelihood ratio
LR (+)	Positive likelihood ratio
M	Mean
P	Percentile
ROC	Receiver operating characteristic curves
SD	Standard deviations
TEM	Technical error of measurement
WC	Waist circumference

## References

[1] Ogden C.L., Carroll M.D., Lawman H.G., Fryar C.D., Kruszon-Moran D., Kit B.K., Flegal K.M. (2016). Trends in Obesity Prevalence among Children and Adolescents in the United States, 1988–1994 through 2013–2014. JAMA.

[2] Expert Panel on Detection, Evaluation, and Treatment of High Blood Cholesterol in Adults (2001). Executive Summary of The Third Report of The National Cholesterol Education Program (NCEP) Expert Panel on detection, evaluation, and treatment of high blood cholesterol in adults (Adult Treatment Panel III). JAMA.

[3] Monasta L., Lobstein T., Cole T.J., Vignerová J., Cattaneo A. (2011). Defining overweight and obesity in pre-school children: IOTF reference or WHO standard?. Obes. Rev..

[4] González-Ruíz K., Correa-Bautista J.E., Ramírez-Vélez R. (2015). Evaluation of the body adiposity index in predicting percentage body fat among Colombian adults. Nutr. Hosp..

[5] Ramírez-Vélez R., Correa-Bautista J.E., Martínez-Torres J., Méneses-Echavez J.F., González-Ruiz K., González-Jiménez E., Schmidt-RioValle J., Lobelo F. (2016). LMS tables for waist circumference and waist–height ratio in Colombian adults: Analysis of nationwide data 2010. Eur. J. Clin. Nutr..

[6] Amato M.C., Guarnotta V., Giordano C. (2013). Body composition assessment for the definition of cardiometabolic risk. J. Endocrinol. Investig..

[7] Von Eyben F.E., Mouritsen E., Holm J., Montvilas P., Dimcevski G., Suciu G., Helleberg I., Kristensen L., von Eyben R. (2003). Intra-abdominal obesity and metabolic risk factors: A study of young adults. Int. J. Obes..

[8] Garn S., Himes J.H. (1991). Implications and applications of subcutaneous fat measurement to nutritional assessment and health risk evaluation. Anthropometric Assessment of Nutritional Status.

[9] González-Jiménez E. (2013). Body composition: Assessment and clinical value. Endocrinol. Nutr..

[10] Sarria A., Garcia-Llop L.A., Moreno L.A., Fleta J., Morellon M.P., Bueno M. (1998). Skinfold thickness measurements are better predictors of body fat percentage than body mass index in male Spanish children and adolescents. Eur. J. Clin. Nutr..

[11] Bedogni G., Lughetti L., Ferrari M., Malavolti M., Poli M., Bernasconi S., Battistini N. (2003). Sensitivity and specificity of body mass index and skinfold thicknesses in detecting excess adiposity in children aged 8–12 years. Ann. Hum. Biol..

[12] Kelly A.S., Dengel D.R., Hodges J., Zhang L., Moran A., Chow L., Sinaiko A.R., Steinberger J. (2014). The relative contributions of the abdominal visceral and subcutaneous fat depots to cardiometabolic risk in youth. Clin. Obes..

[13] Aguirre C.A., Salazar G.D., Lopez de Romaña D.V., Kain J.A., Corvalán C.L., Uauy R.E. (2015). Evaluation of simple body composition methods: Assessment of validity in prepubertal Chilean children. Eur. J. Clin. Nutr..

[14] Freedman D.S., Dietz W.H., Srinivasan S.R., Berenson G.S. (2009). Risk factors and adult body mass index among overweight children: The Bogalusa Heart Study. Pediatrics.

[15] Ali O., Cerjak D., Kent J.W., James R., Blangero J., Zhang Y. (2014). Obesity, central adiposity and cardiometabolic risk factors in children and adolescents: A family-based study. Pediatr. Obes..

[16] Lim S., Meigs J.B. (2013). Ectopic fat and cardiometabolic and vascular risk. Int. J. Cardiol..

[17] LaMonte M.J., Blair S.N. (2006). Physical activity, cardiorespiratory fitness, and adiposity: Contributions to disease risk. Curr. Opin. Clin. Nutr. Metab. Care.

[18] Kromeyer-Hauschild K., Glässer N., Zellner K. (2012). Percentile curves for skinfold thickness in 7- to 14-year-old children and adolescents from Jena, Germany. Eur. J. Clin. Nutr..

[19] Himes J.H., Dietz W.H. (1994). Guidelines for overweight in adolescent preventive services: Recommendations from an expert committee. The Expert Committee on Clinical Guidelines for Overweight in Adolescent Preventive Services. Am. J. Clin. Nutr..

[20] Addo O.Y., Himes J.H. (2010). Reference curves for triceps and subscapular skinfold thicknesses in US children and adolescents. Am. J. Clin. Nutr..

[21] Moreno L.A., Mesana M.I., González-Gross M., Gil C.M., Ortega F.B., Fleta J., Wärnberg J., León J., Marcos A., Bueno M. (2007). Body fat distribution reference standards in Spanish adolescents: The AVENA Study. Int. J. Obes. (Lond.).

[22] Tanner J.M., Whitehouse R.H. (1975). Revised standards for triceps and subscapular skinfolds in British children. Arch. Dis. Child..

[23] Rodríguez-Bautista Y.P., Correa-Bautista J.E., González-Jiménez E., Schmidt-RioValle J., Ramírez-Vélez R. (2015). Values of waist/hip ratio among children and adolescents from Bogotá, Colombia: The FUPRECOL Study. Nut. Hosp..

[24] Aguilar de Plata A.C., Pradilla A., Mosquera M., Gracia de Ramírez A.B., Ortega J.G., Ramírez-Vélez R. (2011). Centile values for anthropometric variables in Colombian adolescents. Endocrinol. Nutr..

[25] Barría R.M., Amigo H. (2006). Nutrition transition: A review of Latin American profile. Arch. Latinoam. Nutr..

[26] Instituto Colombiano de Bienestar Familiar Encuesta Nacional de la Situación Nutricional en Colombia, 2010. http://www.icbf.gov.co/portal/page/portal/PortalICBF/NormatividadC/ENSIN1/.

[27] Prieto-Benavides D.H., Correa-Bautista J.E., Ramírez-Vélez R. (2015). Physical activity levels, physical fitness and scree time among children and adolescents from Bogotá, Colombia: The FUPRECOL Study. Nutr. Hosp..

[28] Ramírez-Vélez R., Rodrigues-Bezerra D., Correa-Bautista J.E., Izquierdo M., Lobelo F. (2015). Reliability of Health-Related Physical Fitness Tests among Colombian Children and Adolescents: The FUPRECOL Study. PLoS ONE.

[29] Departamento Administrativo Nacional de Estadística (DANE) (2007). Los Grupos Étnicos de Colombia en el Censo de 2005.

[30] Marfell-Jones M., Olds T., Stewart A. (2006). International Standards for Anthropometric Assessment.

[31] Moreno L.A., Fleta J., Sarría A., Rodríguez G., Gil C., Bueno M. (2001). Secular changes in body fat patterning in children and adolescents of Zaragoza (Spain), 1980–1995. Int. J. Obes. Relat. Metab. Disord..

[32] Cole T.J., Green P.J. (1992). Smoothing reference centile curves: The LMS method and penalized likelihood. Stat. Med..

[33] Royston P., Wright E.M. (2000). Goodness-of-fit statistics for age-specific reference intervals. Stat. Med..

[34] Cole T.J., Bellizzi M.C., Flegal K.M., Dietz W.H. (2000). Establishing a standard definition for child overweight and obesity worldwide: International survey. BMJ.

[35] Freedman D.S., Katzmarzyk P.T., Dietz W.H., Srinivasan S.R., Berenson G.S. (2009). Relation of body mass index and skinfold thicknesses to cardiovascular disease risk factors in children: The Bogalusa Heart Study. Am. J. Clin. Nutr..

[36] Aristizabal J.C., Barona J., Hoyos M., Ruiz M., Marín C. (2015). Association between anthropometric indices and cardiometabolic risk factors in pre-school children. BMC Pediatr..

[37] Nagy P., Kovacs E., Moreno L.A., Veidebaum T., Tornaritis M., Kourides Y., Siani A., Lauria F., Sioen I., Claessens M. (2014). Percentile reference values for anthropometric body composition indices in European children from the IDEFICS study. Int. J. Obes. (Lond.).

[38] Hirschler V., Molinari C., Maccallini G., Hidalgo M., Gonzalez C. (2016). Waist Circumference Percentiles in Indigenous Argentinean School Children Living at High Altitudes. Child. Obes..

[39] Ma L., Cai L., Deng L., Zhu Y., Ma J., Jing J., Chen Y. (2016). Waist Circumference is Better than Other Anthropometric Indices for Predicting Cardiovascular Disease Risk Factors in Chinese Children-a Cross-Sectional Study in Guangzhou. J. Atheroscler. Thromb..

[40] Gracia-Marco L., Moreno L.A., Ruiz J.R., Ortega F.B., de Moraes A.C., Gottrand F., Roccaldo R., Marcos A., Gómez-Martínez S., Dallongeville J. (2016). Body Composition Indices and Single and Clustered Cardiovascular Disease Risk Factors in Adolescents: Providing Clinical-Based Cut-Points. Prog. Cardiovasc. Dis..

[41] Palczewska I., Niedzwiedzka Z. (2001). Somatic development indices in children and youth of Warsaw. Med. Wieku Rozwoj..

[42] Chrzanowska M., Gołąb S., Żarów R., Sobiecki J., Brudecki J. (2002). The child of Cracow 2000. The Level of the Biological Development in the Cracow Children and Youth.

[43] Ozturk A., Budak N., Cicek B., Mazicioglu M.M., Bayram F., Kurtoglu S. (2009). Cross-sectional reference values for midupper arm circumference, triceps skinfold thickness and arm fat area of Turkish children and adolescents. Int. J. Food Sci. Nutr..

[44] Cicek B., Ozturk A., Unalan D., Bayat M., Mazicioglu M.M., Kurtoglu S. (2014). Four-site skinfolds and body fat percentage references in 6-to-17-year old Turkish children and adolescents. J. Pak. Med. Assoc..

[45] Brannsether B., Roelants M., Bjerknes R., Júlíusson P.B. (2013). References and cutoffs for triceps and subscapular skinfolds in Norwegian children 4–16 years of age. Eur. J. Clin. Nutr..

[46] Khadilkar A., Mandlik R., Chiplonkar S., Khadilkar V., Ekbote V., Patwardhan V. (2015). Reference centile curves for triceps skinfold thickness for Indian children aged 5 to 17 years and cut offs for predicting risk of childhood hypertension: A multi-centric study. Indian Pediatr..

[47] Palmer B.F., Clegg D.J. (2015). The sexual dimorphism of obesity. Mol. Cell. Endocrinol..

[48] Khoury M., Manlhiot C., Gibson D., Chahal N., Stearne K., Dobbin S., McCrindle B.W. (2016). Universal screening for cardiovascular disease risk factors in adolescents to identify high-risk families: A population-based cross-sectional study. BMC Pediatr..

[49] Anderson L.N., Lebovic G., Hamilton J., Hanley A.J., McCrindle B.W., Maguire J.L., Parkin P.C., Birken C.S., TARGet Kids Collaboration (2016). Body Mass Index, Waist Circumference, and the Clustering of Cardiometabolic Risk Factors in Early Childhood. Paediatr. Perinat. Epidemiol..

[50] Stettler N., Zomorrodi A., Posner J.C. (2007). Predictive value of weight-for-age to identify overweight children. Obesity.

